# A Novel Enzyme-Based SPR Strategy for Detection of the Antimicrobial Agent Chlorophene

**DOI:** 10.3390/bios11020043

**Published:** 2021-02-09

**Authors:** Gabriela Elizabeth Quintanilla-Villanueva, Donato Luna-Moreno, Edgar Allan Blanco-Gámez, José Manuel Rodríguez-Delgado, Juan Francisco Villarreal-Chiu, Melissa Marlene Rodríguez-Delgado

**Affiliations:** 1 Laboratorio de Biotecnología, Facultad de Ciencias Químicas, Universidad Autónoma de Nuevo León, Av. Universidad S/N Ciudad Universitaria, San Nicolás de los Garza C.P. 66455, Nuevo León, Mexico; gabriela.quintanillavl@uanl.edu.mx (G.E.Q.-V.); edgar.blancogmz@uanl.edu.mx (E.A.B.-G.); juan.villarrealch@uanl.edu.mx (J.F.V.-C.); 2 Centro de Investigación en Biotecnología y Nanotecnología (CIByN), Facultad de Ciencias Químicas, Universidad Autónoma de Nuevo León. Parque de Investigación e Innovación Tecnológica, Km. 10 Autopista al Aeropuerto Internacional Mariano Escobedo, Apodaca C.P. 66629, Nuevo León, Mexico; 3 Centro de Investigaciones en Óptica AC, Div. de Fotónica, Loma del Bosque 115, Col. Lomas del Campestre, León C.P. 37150, Guanajuato, Mexico; 4 Tecnológico de Monterrey, School of Engineering and Sciences, Av. Eugenio Garza Sada Sur No. 2501, Col. Tecnológico, Monterrey C.P. 64849, Nuevo León, Mexico; jmrd@tec.mx

**Keywords:** SPR biosensor, enzyme, laccase, chlorophene, emerging pollutant, water sample

## Abstract

Chlorophene is an important antimicrobial agent present in disinfectant products which has been related to health and environmental effects, and its detection has been limited to chromatographic techniques. Thus, there is a lack of research that attempts to develop new analytical tools, such as biosensors, that address the detection of this emerging pollutant. Therefore, a new biosensor for the direct detection of chlorophene in real water is presented, based on surface plasmon resonance (SPR) and using a laccase enzyme as a recognition element. The biosensor chip was obtained by covalent immobilization of the laccase on a gold-coated surface through carbodiimide esters. The analytical parameters accomplished resulted in a limit of detection and quantification of 0.33 mg/L and 1.10 mg/L, respectively, fulfilling the concentrations that have already been detected in environmental samples. During the natural river’s measurements, no significant matrix effects were observed, obtaining a recovery percentage of 109.21% ± 7.08, which suggested that the method was suitable for the fast and straightforward analysis of this contaminant. Finally, the SPR measurements were validated with an HPLC method, which demonstrated no significant difference in terms of precision and accuracy, leading to the conclusion that the biosensor reflects its potential as an alternative analytical tool for the monitoring of chlorophene in aquatic environments.

## 1. Introduction

Emerging pollutants are persistent chemicals in the environment, classified as pharmaceutical compounds or their metabolites (human and veterinary drugs). These include personal care products (e.g., disinfectants, fragrances, insect repellents, cosmetics and sunscreens) and endocrine disrupting compounds (e.g., bisphenol A, triclosan and pesticides) [1]. In particular, halogenated phenolic compounds comprise the vast majority of the active ingredients employed in the manufacture of personal care products [2]. In this sense, chlorophene (4-chloro-2-(phenylmethyl)phenol) is an antimicrobial agent widely applied in disinfectants for cleaning activities and for farming, industrial and household environments [3,4], as well as preservatives in cosmetics and wood [5]. According to the Environmental Protection Agency (EPA), chlorophene has been included in the list of priority toxic pollutants [6,7]. It has been related to mutagenic effects in mammals [5], fertility alterations and kidney damage through prolonged exposure [4]. The occurrence of chlorophene (CP) has been reported in water [7] and soil [8]. For example, concentrations up to 0.13 mg/L of CP were detected in a backwater stream in Kerala (India) [7]. Meanwhile, 50 mg/L was quantified in activated sludge sewage, and 10 μg/L was quantified in treatment plant effluent [9].

These micropollutants enter the environment through anthropogenic pathways [7]. A trace amount results in ecological risks, such as biomagnification along the food chain due to accumulation in organisms by hydrophobic properties [3]. Such is the case of CP’s occurrence in male bream bile from the Dommel river (7 μg/mL) [10]. The presence of this emerging pollutant has been commonly detected by high performance liquid chromatography mass spectrometry (HPLC-MS) [4] and gas chromatography mass spectrometry (GC-MS) [3], powerful analytical methods for detecting and quantifying trace amounts of compounds. For instance, Rayaroth et al. (2015) [7] identified the presence of chlorophene in a backwater stream using the liquid chromatography quadrupole time of flight MS (LC-QTOF-MS), with a C18 column set at 35 °C and a gradient elution of acetonitrile:formic acid in water (0.1%). Meanwhile, Chen et al. (2018) [11] established the quantification of CP using an HPLC instrument with a UV absorbance detector, an SB-C18 column and a mixture of formic acid as a mobile phase and methanol. On the other hand, the use of the GC-MS technique has also been reported, employing a 5% phenyl methyl siloxane capillary column, splitless injection at 250 °C, an oven temperature from 70 to 280 °C (10 °C/min) and helium as the carrier gas [3]. It is worth highlighting that in prior chromatography−mass spectrometry analysis, sample pretreatment needed to be performed, commonly a purified process by solid-phase extraction (SPE) using cartridges [11] or solvent extraction followed by a concentration step [3]. Consequently, the time-consuming sample preparation and lab environments’ restrictions remain significant drawbacks that limit chromatographic techniques. Thus, there is an increasing interest in developing new analytical tools that provide fast, sensitive, and in situ measurements, such as biosensor systems. In this sense, the surface plasmon resonance (SPR) technique has had significant relevance in the environmental field. For example, it was employed in the detection of endocrine disruptors (estrogen [12] and bisphenol A [13]), organophosphate pesticides like chlorpyrifos [14] and industrial pollutants such as polychlorinated biphenyls [15]. Nevertheless, no attempt to use biosensors to detect CP has been explored.

On the other hand, diverse treatment processes have been applied to remove CP, such as MnO_2_ oxidation, persulfate treatment and ozonation [11]. However, the use of laccase enzymes in removing chlorophene and dichlorophene is worth noticing [16]. Laccases are phenoloxidases produced in extracellular form by a diverse variety of organisms, from higher plants to fungi [17,18] and bacteria [19]. These enzymes catalyze the oxidation of organic compounds by the concomitant reduction of oxygen [20]. In particular, the removal of CP by laccase catalysis was demonstrated by Shi et al. (2016), suggesting a direct polymerization as the principal mechanism for elimination [16].

Therefore, this work establishes the immobilization of laccase enzymes for their use as a receptor in the detection of chlorophene using an SPR technique. The use of enzymes as recognition elements is very uncommon in SPR techniques [21,22], considering that most applications rely on antigen–antibody interactions [23], aptamer recognition [24,25] and nucleic acid hybridization [26]. In particular, studies of laccase as a bioreceptor in SPR are very scarce [27]. The proposed enzyme-based SPR biosensor’s analytical parameters, such as the limit of detection, sensitivity and working range, were studied. Finally, fortified real water samples were analyzed by the SPR biosensor, and the results obtained were compared in terms of accuracy and precision against a well-known HPLC method.

## 2. Materials and Methods

### 2.1. Reagents

All the chemical compounds employed in the enzyme immobilization and the biosensing process, such as 16-mercaptohexadecanoic acid (MHDA), 11-mercaptoundecanol (MUD), ethanolamine hydrochloride, N-hydroxysuccinimide (NHS) and 1-ethyl-3-(3-dimethylamino-propyl) carbodiimide hydrochloride (EDC)) were purchased from Sigma-Aldrich (St. Louis, MO, USA). The laccase enzymes (*Rhus vernicifera*) and salts employed in buffers were purchased from Sigma-Aldrich (St. Louis, MO, USA).

HPLC analysis was performed by employing a Zorbax ODS C18, 25 cm × 4.6 mm, 5 µm column, which was purchased from SUPELCO Analytical (St. Louis, MO, USA) with an HPLC system model YL9100 (Younglin Instrument Co., Ltd., Gyeonggi-do, Korea). The HPLC-grade acetonitrile and water were purchased from Merck (Darmstadt, Germany).

The standards of chlorophene (analytical grade, Sigma-Aldrich, Mexico City, Mexico) were prepared as stock solutions (10 mg/mL) in ethanol:water (90:10, %*v*/*v*) 96 and completed at 10 mL with ultrapure water. From these stock solutions, working solutions were prepared by serial dilution in a water:phosphate-buffered saline solution with a pH of 7.3 (90:10, %*v*/*v*) in a concentration range from 0 to 10 mg/L.

Real water samples were obtained from a river and filtered with Whatman grade 40 filter as the only pretreatment. Then, one level of fortification was prepared by spiking the river samples with chlorophene at a concentration of 3 mg/L, followed by its analysis by HPLC and the SPR technique.

### 2.2. Sample Collection and Characterization

Water samples were collected from a river located in León city, Guanajuato-México (21°09′54.0′′ N, 101°43′30.6′′ W). Sample collection was performed following the Mexican standards established in NOM-230-SSA1-2002 [28]. Briefly, at the sampling site, water samples were collected in polyethylene bottles (pre-rinsed with distillate water). The temperature and pH were measured in the area with a multiparameter probe (WTW Multi 350i). Analyses of the sulfate, total alkalinity and acidity were performed according to the Mexican standards NMX-AA-036-SCFI-2001 [29], as well as the hardness following the methods of NMX-AA-072-SCFI-2001 [30] and the chlorides according to NMX-AA-073-SCFI-2001 [31]. The total organic carbon (COT) and heavy metals were measured by a Shimadzu analyzer by catalytic oxidation of combustion (TOC-L, Shimadzu, Kyoto, Japan) and atomic absorption (Thermo Jarrell Ash Scan1, Franklin, MA, USA), respectively, under NMX-AA-051-SCFI-2016 [32].

### 2.3. Cr/Au Thin Film Deposition

Homogeneous thin films of Cr/Au were deposited on thin glass substrates by electron gun evaporation, following the method described by Luna-Moreno [33]. Briefly, the chromium layer was evaporated up to a 3 nm thickness. Then, a gold film of 50 nm was deposited by thermal evaporation at a rate of 5 Å/s and 8 × 10^−6^ mbar. The thickness of the thin films was evaluated by employing a quartz crystal microbalance thickness monitor (Leybold Inficon XTC/2 Depositions controllers).

### 2.4. SPR Instrumentation

**Figure 1:**
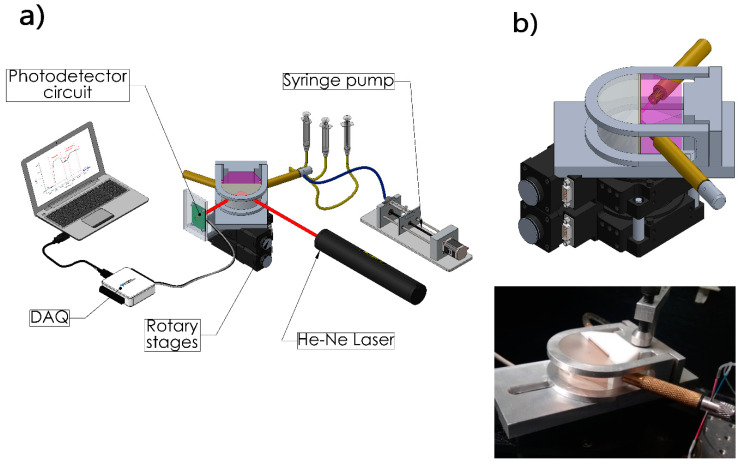
(**a**) Surface plasmon resonance (SPR) setup and (**b**) scheme of the measuring cell.

The SPR setup was a homemade platform described previously by Sánchez-Alvarez et al. (2018) [34], based on a Kretschmann configuration and comprising two stacked rotation plates, configured for synchronized movement according to a θ-2θ system by a stepper motor. The measuring cell in the SPR system consisted of a sandwich configuration integrated by a Teflon cell, a gold thin film chip and a hemicylindrical BK7 glass prism. The substrate’s glass surface was optically coupled to the prism using an oil matching index (n = 1.51). Meanwhile, the chip’s gold-coated surface was facing against the flow of the Teflon cell, which had an inlet and outlet that allowed the solutions to come in contact with the gold through its inner channel (Figure 1). Our design allowed for adjusting to customized measurement chips (different size and thickness), depending on the desired application compared to commercial cells.

The chemical solutions continuously flowed through the measuring cell via a syringe pump (Legato 100) at a rate of 30 μL min^−1^. Furthermore, a photodetector (Hamamatsu, model S1226-8Bk) was used to capture the reflected light of a He-Ne laser (Uniphase mod. 1101P) that passed through the prism.

### 2.5. Enzymatic Activity

The laccase enzymatic activity was measured through the spectrophotometric UV-Vis assay established by Zhang et al. (2018) [35]. For the assay, 200 µL of the enzyme was added to a reaction mixture (2 mL) containing 10 mM of 2,2′-azino-bis(3-ethylbenzothiazoline-6-sulfonic acid) (ABTS) in a 0.1 M sodium acetate buffer with a pH of 4.5. The reaction occurred at room temperature, and absorbance changes were recorded at 420 nm in a UV-Vis spectrophotometer (Cary 50, Varian Inc., Palo Alto, CA, USA).

Enzyme activity was expressed as a function of the amount of enzyme necessary to produce 1 µM of product per minute (U) and was calculated by the following equation:

$$Activity=\frac{\left[\left(\frac{\Delta Abs}{min}\right)\times V_{t}\right]}{\varepsilon \times 10^{4}\times 1\times Vm}$$

where Δ*Abs* is the change in absorbance, *V_t_* is the total volume of the cell, ε is the molar extinction coefficient of ABTS (36,000 M^−1^cm^−1^) at 420 nm and *Vm* is the volume of the laccase sample [35].

### 2.6. Enzyme Bioreceptor: Chip Functionalization and Laccase Immobilization

Before the immobilization process, a functionalization treatment on the gold substrate (50 nm chips) was performed. Briefly, the gold chips were cleaned by consecutive immersion in acetone and ethanol (30 s in each solvent) and then dried with air. Then, the clean chips were immersed for 12 h at room temperature in a solution of alkanethiols MHDA:MUD (250 µM in ethanol) [36]. The sulfur group from the alkanethiols bound to the gold. Meanwhile, the free carboxylic group on the other end of the chain served as the binding site for the further immobilization of the enzyme.

Once the chip’s surface was functionalized with the alkanethiols, the carboxylic groups were activated using the EDC/NHS crosslinkers [37]. A solution of EDC/NHS (EDC 0.2 M/NHS 0.05 M) in an MES buffer (100 mM, 500 mM NaCl, pH 5.0) was flowed on the gold surface, allowing the formation of carbodiimide esters. Then, a solution of 200 U mg-1 of laccase was injected. By creating an amide bond between the amino acids of the enzyme and the activated carboxylic terminal group, the laccase’s attachment occurred, ending the immobilization process. Finally, the remaining active esters were deactivated with a solution of ethanolamine (1 M, pH 8.5), preventing unspecific bindings. Once the immobilization process concluded, a washing step was performed, flowing over the sensor surface a phosphate buffer solution (PBS) to remove non-bonded molecules.

### 2.7. SPR Measurements: Chlorophene Detection

Once the chip was mounted on the SPR setup, the working angle was established at the slope’s midpoint, formed in the SPR curve (approaching the critical angle). At this point, greater sensitivity to changes in light intensity, caused by the interaction of the receptor with the analyte, was achieved.

The analysis of chlorophene (CP) was performed by a direct enzyme–substrate assay, where the immobilized laccase enzymes catalyzed the oxidation reaction of CP in the sample. The obtained signals (enzyme–substrate binding) were directly proportional to the concentration of the analyte in the samples, since a shift in the conformation of the enzyme occurred as a result of CP binding in the active site of the laccase, observed as a change in the refractive index measured by the photodetector [38]. The PBS buffer was set as a running solution during the measurement process, and the samples containing CP (0–10 mg/mL) were flowed at 30 µL/min over the sensor surface. The sensor surface was then washed with a PBS buffer injection to remove weakly bound CP from the biofunctionalized chip. Finally, a regeneration solution (NaOH 10 mM) was injected for 20 s to release the bioreceptor and prepare it for a new measurement cycle. All measurements were performed in triplicate. The obtained average SPR signals were plotted as a CP concentration function in the sample. The calibration curve generated was employed to establish the analytical parameters of the biosensor. The sensitivity of the method corresponded to the slope of the curve. The detection limit was evaluated as three times the standard deviation of the baseline, while the limit of quantitation was 10 times the standard deviation. The recovery and reproducibility of the analytical procedure were established using spiked real samples. The determination of the recovery and precision of the SPR was also performed on natural samples, evaluating possible matrix effects.

### 2.8. HPLC Measurements

The HPLC analysis (YL9300, Thermofisher-USA) was performed according to the method previously described [4]. Briefly, a solution of acetonitrile:water (85:15) was employed as a mobile phase at a flow rate of 1 mL/min, using a Zorbax ODS C18, 25 cm × 4.6 mm (5 µm particle size) column and a UV detector at 290 nm [7]. The calibration standards of the CP stock were prepared in ethanol from a stock solution of 100 mg/L (working range from 0–10 mg/L). The recovery and reproducibility of the analytical procedure were established using spiked real samples.

## 3. Results and Discussion

### 3.1. Chip Functionalization and Laccase Immobilization

**Figure 2:**
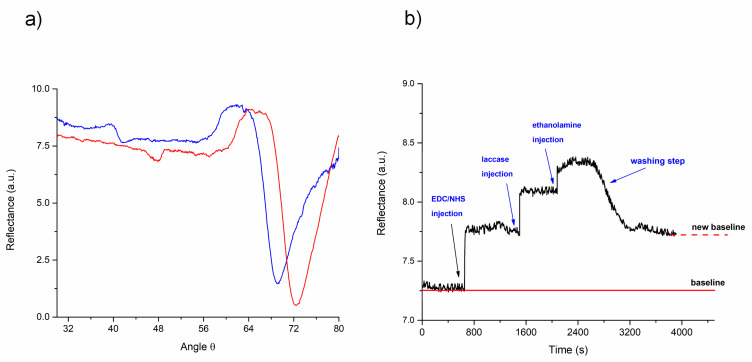
(**a**) Reflectance spectra obtained by the angular sweep of a sensor chip with alkanethiols (blue line) and after immobilization of the laccase (red line). (**b**) SPR sensorgram of laccase immobilization.

The immobilization process of the enzymes was initiated with the functionalization of a gold-coated chip. After 12 h of incubation, the binding alkanethiols were activated through the EDC/NHS cross-linkers, forming an amide bond that attached to the laccase enzymes. Figure 2a shows the critical angle displacement of the SPR curve of the alkanethiol-coated chip (blue line) in comparison with the SPR curve after laccase immobilization occurred (red line), showing a shift of 3.2 degrees (from 69.2° to 72.4°) in the resonance angle.

The shift was attributed to the increase of the mass density due to the bound enzymes on the surface. In this sense, several studies have quantified the immobilization yield on a surface through a conversion factor of 1 ng/mm^2^ of biomolecules or protein. The conversion factor was related to a change of 0.1° in the SPR angle (1000 refractive units) [39,40,41]. Therefore, the angle displacement obtained in this study would represent a density of 32 ng/mm^2^ of enzyme onto the gold-coated chip.

The immobilization process was also monitored in real time at a fixed angle of 66.8° (highest sensitivity from the SPR curve slope), obtained by the angular sweep of the immobilized SPR chip (Figure 2a). The sensorgram obtained from the immobilization process in-flow is observed in Figure 2b. The increase of the SPR baseline signal was notable after the subsequent addition of the EDC/NHS crosslinkers, the laccase enzyme and the ethanolamine. However, after the washing step, the signal decreased, suggesting the removal of those molecules weakly bonded on the surface. Once the washing process concluded, it was noticeable that the SPR signal was higher than the initial baseline (prior immobilization), inferring the successful linkage of the molecules that remained on the surface. These results agree with the ones obtained by the angular swept measurements (Figure 2a).

### 3.2. SPR Measurements: Chlorophene Detection

A direct enzyme–substrate assay was performed to detect chlorophene ranging from 0–10 mg/mL, using an SPR gold-coated chip immobilized with laccase enzymes. In this biosensor, the immobilized laccase catalyzed the oxidation reaction of CP in the sample, observed as a change in the refractive index (SPR signal) due to a shift in the conformation of the enzyme as a result of the concentration of analyte binding to its active site [38]. The active site of the laccase enzymes comprised four copper atoms (type I, type II and two type III copper atoms) [42]. The enzyme’s catalytic mechanism involved the substrate oxidation in the type I copper site, followed by an internal electron transfer from the reduced type I atoms to the type II and type III trinuclear cluster, where the reduction of dioxygen to water occurred [42].

**Figure 3:**
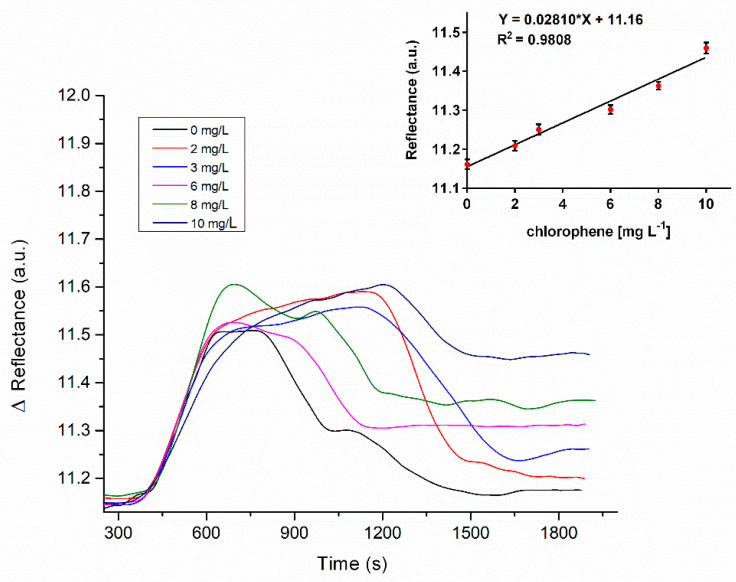
SPR sensorgrams for chlorophene (CP) detection at different concentrations and calibration curves in PBS (*n* = 3).

According to Enguita et al. (2004) [43], apolar groups in chemical structures are attached to a hydrophobic binding site in laccase, which is located in proximity to the type I Cu site of the enzyme through the His497 residue (one of the type I copper ligands). In this sense, the phenyl group of the chlorophene molecule might present a close approach toward the aromatic ring of the His497 residue in laccase, favoring the electron transfer from CP (oxidation process) to the type I copper and subsequent internal transfer to the trinuclear cluster [43]. Jabbari et al. (2017) [27] reported this electron transfer mechanism during the study to detect the bromocriptine drug by the SPR technique using laccase from Bacillus sp. HR03 [27]. The SPR signal value at the plateau (saturation in the binding event) obtained from the sensorgrams was plotted as a function of the CP concentrations to generate a calibration curve (Figure 3).

The analytical parameters obtained in this study are summarized in Table 1. The results obtained meet the concentration detected in a backwater stream in Kerala (India) [7] and in activated sludge sewage [9] by HPLC-MS and GC-MS. Additionally, the immobilized enzyme withstood 35 regeneration cycles by using 0.1 M NaOH before any significant loss of recognition capacity was observed. Currently, there is a lack of biosensors that address the detection of CP, since the analytical tools have been mainly limited to chromatographic techniques. Thus, this research represents the first approach to the fabrication of a robust SPR platform for the routine monitoring of chlorophene in water.

### 3.3. Evaluation of SPR Performance with River Water: Study of Matrix Effects

**Figure 4:**
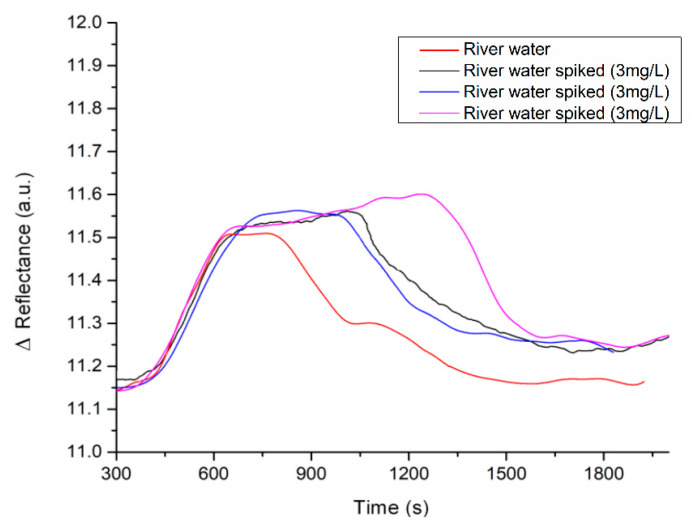
Evaluation of nonspecific signals due to matrix effects from the river water.

Certain components in the real sample matrix could lead to false positives or unspecific bindings that interfere with bioreceptor recognition [44]. Thus, this is a critical issue to determine the performance of a method during the analysis of real samples. Possible matrix effects due to river water composition need to be evaluated. Thus, a natural water sample was injected as a control. Apparently, no significant SPR signal was shown in the preliminary sensitivity assay result with the river water, suggesting that no significant enzyme–substrate reaction occurred. However, it is essential to perform a selectivity assay in the presence of related compounds (interferences) to confirm the method’s feasibility for the selective detection of chlorophene. Then, spiked river samples with 3 mg/mL of CP (triplicate) were measured, obtaining nearly identical SPR responses among them under these conditions (see Figure 4).

The characterization of the river samples can be observed in Table 2, where it is worth noticing that the concentration of organic matter did not represent a considerable effect on the recovery percentage analysis, showing a recovery percentage of 109.21% under the method conditions (see Table 3). No significant differences were found between the theoretical concentration and the one obtained experimentally (*p* = 0.05, *n* = 3).

On the other hand, the laccase enzymes were stable under the concentration of dissolved chlorides and the alkaline pH, since those are conditions that tend to affect the enzymatic activity of these enzymes [45,46].

### 3.4. Comparison of SPR Protocol with the HPLC Method

Samples spiked in a range from 0 to 30 mg/L were analyzed by HPLC. The linear regression analysis provided a correlation of 0.9995, a limit of detection (LOD) of 0.07 mg/L and a limit of quantification (LOQ) of 0.22 mg/L, with an operating range of 0–30 mg/L.

Then, the river samples spiked with chlorophene at concentrations of 3 mg/L were analyzed. A Student’s *t*-test with 95% confidence was evaluated, and no significant discrepancies were found when comparing the SPR and HPLC methods, indicating excellent agreement between the techniques (Table 3).

## 4. Conclusions

This work established the use of a homemade SPR biosensor based on using laccase enzymes as a bioreceptor for the real-time detection of the hazardous antimicrobial chlorophene in real waters. To the best of our knowledge, this study is the first attempt to develop a biosensor to detect chlorophene. The analytical parameters accomplished by the SPR biosensor fulfilled the concentrations that have already been detected in natural water samples. The biosensor method resulted in a limit of detection and quantification of 0.33 mg/L and 1.10 mg/L, respectively. Although no apparent matrix effects were detected in the analytical response of the SPR measurements of the river samples, it is essential to perform a selectivity assay to confirm the method’s feasibility in the selective detection of chlorophene. Furthermore, the method’s reliability could be improved by analyzing certified samples to complement fortified natural water results. Finally, the comparison of SPR measurements with an HPLC conventional method demonstrated no significant difference in precision and accuracy. These results show a considerable advantage, due to its lack of a pretreatment process, which is required by traditional techniques, suggesting a suitable and straightforward analysis of this contaminant in natural water.

## Figures and Tables

**Table 1:** Analytical parameters of SPR-based biosensors for chlorophene detection.

Bioreceptor	LOD (mg mL^−^^1^)	LOQ (mg mL^−^^1^)	Sensitivity (Reflectance/mg mL^−^^1^)	Dynamic Range (mg mL^−^^1^)
Laccase enzyme	0.33 ± 0.01	1.1 ± 0.01	0.0281 ± 0.0001	0–10

**Table 2:** Components and significant ion concentrations (mg/L) in the river water samples.

pH	Total Hardness	Calcium-Based Hardness	Magnesium-Based Hardness	Chloride	Sulfates	Total Organic Carbon	Inorganic Carbon	As	Cu	Pb
8.05 ± 0.07	220.7 ± 6.4	151.3 ± 10.3	69.5 ± 3.9	10.99 ± 0.49	32.11 ± 1.85	23.66	60.75	˂0.003	˂0.1	˂0.2

**Table 3:** Determination of spiked river samples by the SPR and HPLC methods (*n* = 3).

Fortification Level (mg mL^−1^)	SPR Method		HPLC Method	
3	Mean (mg mL^−1^)	Recovery (%)	Mean (mg mL^−1^)	Recovery (%)
	3.28 ± 0.27	109.21 ± 7.08	3.04 ± 0.01	101.33 ± 3.55
